# Thromboembolic Risk After Total Hip Replacement Versus Hemiarthroplasty in Femoral Neck Fracture Patients: A Systematic Review and Meta-Analysis

**DOI:** 10.3390/medicina61111929

**Published:** 2025-10-28

**Authors:** Ibrahim A. Hakami, Mohammed A. Altammar, Shafi A. Alaklabi, Meshari M. Alotaibi, Saleh N. Almunyif, Mohammed I. Alshuwaier, Sultan T. Alobaysi, Sultan S. Aldalbahi, Abdullah H. Alotaibi, Mohammed M. Alotaibi, Omar S. Alobaysi, Moath T. Aladhyani, Mohammad A. Jareebi

**Affiliations:** 1 Department of Orthopedics and Trauma Surgery, Shaqra University, Shaqra 11961, Saudi Arabia; ihakami@su.edu.sa; 2 Faculty of Medicine, Shaqra University, Dawadmi 11911, Saudi Arabia; 3 Family and Community Medicine Department, Faculty of Medicine, Jazan University, Jazan 45142, Saudi Arabia

**Keywords:** femoral neck fracture, total hip replacement, hemiarthroplasty, venous thromboembolism (VTE), deep vein thrombosis (DVT), pulmonary embolism (PE), ischemic stroke, systematic review, meta-analysis

## Abstract

*Background and Objectives*: Femoral neck fractures are common among elderly patients and are typically managed surgically to restore mobility and reduce complications. Total Hip Replacement (THR) and Hemiarthroplasty (HA) are standard interventions. While both procedures are widely used, the comparative risks of thromboembolic complications remain unclear. This study aimed to systematically compare the risk of Venous Thromboembolism (VTE), including Deep Vein Thrombosis (DVT), Pulmonary Embolism (PE), and Arterial Thromboembolic events in patients undergoing THR versus hemiarthroplasty for femoral neck fractures. *Materials and Methods*: A comprehensive literature search was conducted in PubMed, Web of Science, Google Scholar, and OVID-Medline for studies published between 2000 and 2024. Eligible studies included patients aged ≥50 years with femoral neck fractures treated with THR or Hemiarthroplasty and reported thromboembolic outcomes. Risk of bias was assessed using the Newcastle–Ottawa Scale and the RoB 2 tools. Meta-analysis was performed using RevMan software (Version 5.4). *Results*: Twelve studies were included in the systematic review, and ten in the meta-analysis, encompassing over 160,000 patients. THR was associated with a significantly increased risk of DVT (RR = 1.53; 95% CI: 1.40–1.68, *p* = 0.00001) and combined VTE (RR = 1.48; 95% CI: 1.36–1.61, *p* = 0.00001) compared to HA. No significant difference was observed in PE risk. Interestingly, THR was linked to a lower risk of Arterial Thromboembolic events, such as Ischemic Stroke. *Conclusions*: Compared with Hemiarthroplasty, THR increases the risk of VTE, including DVT, with no increased risk of PE. Surgical decisions should be guided by individual patients’ risk factors for thrombotic and cardiovascular events.

## 1. Introduction

The femoral neck is a narrow area connecting the femur’s head to its shaft, transmitting forces between the hip joint and the thigh bone. Fractures capsulae, known as intracapsular hip fractures, are common and serious in the elderly, as they occur within the joint capsule and can severely affect mobility and quality of life. As individuals age, the natural degeneration of bone density and overall musculoskeletal strength makes them increasingly vulnerable to such injuries [1]. Femoral neck fractures are a growing global health concern, currently affecting around 1.66 million people annually. With increasing life expectancy and an aging population, this number is expected to rise to approximately 6.26 million cases by 2050 [2]. While falls remain the most common cause of femoral neck fractures, several other risk factors play a role in their occurrence. One of the primary contributing factors is decreased bone mineral density, commonly seen in individuals with osteoporosis. This condition leads to brittle bones that are more likely to fracture under stress. Elderly individuals are more likely to sustain femoral neck fractures even from low-impact incidents such as minor slips or falls [1,3]. Prolonged use of corticosteroids, which weaken bone structure, along with a sedentary lifestyle that reduces muscle mass and balance, significantly increases the risk of femoral neck fractures. Clinically, most of these fractures are displaced, meaning the broken bone ends are misaligned and typically require surgical treatment. The two main surgical options are Total Hip Replacement (THR), which replaces both the femoral head and the hip socket, and Hemiarthroplasty, which replaces only the femoral head and neck while preserving the acetabulum [4]. Each surgical approach has its advantages and limitations. Some clinicians prefer THR because it is associated with better functional outcomes, improved pain control, and a reduced likelihood of requiring future revision surgeries. However, it is not without risks—it carries a higher chance of dislocation compared to Hemiarthroplasty. In contrast, Hemiarthroplasty tends to have a lower dislocation rate [5]. Although both procedures are widely practiced, the comparative risk of thromboembolism remains unclear. While some studies suggest a higher thromboembolic risk with THR, others report no significant difference, leading to clinical uncertainty. The choice of surgical treatment should be tailored to the individual patient, considering their age, overall health, activity level, and comorbidities. This study aims to compare the risk of venous thromboembolism (VTE), including deep vein thrombosis (DVT) and pulmonary embolism (PE), as well as the risk of arterial thromboembolic events (ATE), including myocardial infarction (MI) and ischemic stroke, between total hip replacement (THR) and hemiarthroplasty (HA) in patients with femoral neck fractures. The objectives are to systematically review the available literature on thromboembolic complications following THR compared to hemiarthroplasty, conduct a meta-analysis to estimate pooled risk ratios for each procedure, and provide evidence to support clinicians in selecting the most appropriate surgical approach with improved understanding of thromboembolic risks.

## 2. Materials and Methods

### 2.1. Literature Search and Registration

This systematic review followed the Preferred Reporting Items for Systematic Reviews and Meta-Analyses (PRISMA) guidelines [6], and the protocol was registered with the Prospective Register of Systematic Reviews (PROSPERO, Centre for Reviews and Dissemination, University of York, York, UK) (Registration ID: CRD420251039970). A comprehensive search was conducted across multiple electronic databases, including PubMed (National Center for Biotechnology Information, National Library of Medicine, Bethesda, MD, USA), Google Scholar (Google LLC, Mountain View, CA, USA), Web of Science (Clarivate Analytics, Philadelphia, PA, USA), and OVID-Medline (Ovid Technologies, Wolters Kluwer, New York, NY, USA) for articles published from 2000 to 2024. We employed comprehensive search terms: (“Femoral Neck Fractures” OR “Hip Fractures”) AND (“Total Hip Arthroplasty” OR “Hemiarthroplasty”) AND (“Venous Thromboembolism” OR “Deep Vein Thrombosis” OR “Pulmonary Embolism” OR “Myocardial Infarction” OR “Stroke”).

### 2.2. Study Selection

Studies were selected based on predefined inclusion and exclusion criteria:

#### 2.2.1. Inclusion Criteria

We included studies that examined elderly patients aged 50 years or older diagnosed with femoral neck fractures. Studies compared outcomes between patients treated with total hip replacement (THR) and those treated with hemiarthroplasty. Studies were required to report thromboembolism-related outcomes, including deep vein thrombosis (DVT), pulmonary embolism (PE), myocardial infarction (MI), and ischemic stroke. Only randomized controlled trials (RCTs) and cohort studies published in English were considered for inclusion.

#### 2.2.2. Exclusion Criteria

We excluded studies involving pathological fractures, polytrauma patients, bedridden individuals, and patients with pre-existing DVT or PE unless these conditions were reported separately. Studies were also excluded if they investigated other surgical interventions (e.g., internal fixation), lacked a direct comparison between THR and hemiarthroplasty, or did not report thromboembolic outcomes or sufficient data. In addition, case reports, case series, expert opinions, reviews, and animal or cadaveric studies were not included.

### 2.3. Data Extraction

After duplicate removal using Rayyan AI, two independent reviewers screened the titles and abstracts. Full-text articles were then reviewed independently by two other reviewers. Disagreements were resolved by a third reviewer. Data was extracted independently by three reviewers, covering: study characteristics, patient demographics, interventions, follow-up duration, and thromboembolic outcomes. Discrepancies were resolved through discussion with two senior reviewers. All extracted data were cross verified for accuracy. Primary outcomes included venous thromboembolism events (VTE), such as deep vein thrombosis (DVT) and pulmonary embolism (PE). DVT was diagnosed using duplex ultrasonography or venography, and PE was confirmed by CTPA or ventilation–perfusion (V/Q) scanning. The secondary outcomes included arterial thromboembolic events (ATE), such as myocardial infarction (MI) and ischemic stroke.

Quality and Risk of bias were assessed according to study type. For Randomized Controlled Trials (RCTs), we used the RoB 2 tool [7], evaluating selection, performance, and other biases. Each RCT was categorized as having a low, high, or unclear risk of bias. Cohort studies were evaluated using the Newcastle–Ottawa Scale (NOS) [8], considering selection, comparability, and outcome domains. Studies scoring 7–9 stars were deemed low risk, 4–6 stars moderate risk, and 0–3 stars high risk. Two reviewers independently assessed quality, resolving disagreements through consensus.

### 2.4. Statistical Analysis

Statistical analyses were conducted using Review Manager (RevMan, version 5.4) software. For dichotomous outcomes, risk ratios (RRs) with 95% confidence intervals (CIs) were calculated. Outcomes included venous thromboembolism (VTE) events (overall VTE, DVT, PE) and arterial thromboembolism (ATE) events (MI, ischemic stroke). Heterogeneity was assessed using Chi-square (χ^2^) and quantified by I^2^ statistics. An I^2^ < 50% indicated low heterogeneity, and a fixed-effects model was applied. If I^2^ exceeded 50%, a random-effects model would have been used; however, most outcomes showed low to moderate heterogeneity, supporting fixed-effects modeling. In analyses with high heterogeneity, sensitivity analysis will be considered. Subgroup analyses based on specific thromboembolic outcomes (VTE, DVT, PE, MI, stroke) were conducted. Subgroup differences were also tested. A *p*-value < 0.05 was considered statistically significant. Results were presented graphically using forest plots with pooled estimates and 95% CI.

## 3. Results

### 3.1. Characteristics of Included Studies

**Figure 1:**
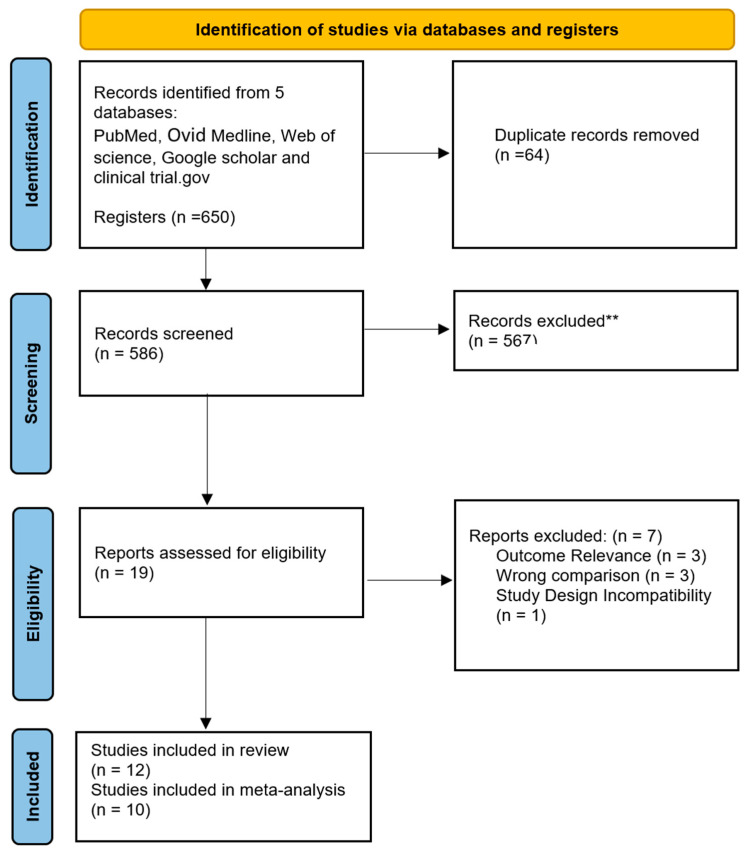
PRISMA Flow Diagram.

The initial search across four databases yielded 650 records. After removing 64 duplicates, 564 unique articles remained for title and abstract screening. Based on the screening, 19 articles were selected for full-text review. Following full-text assessment, 12 studies met all inclusion criteria for the systematic review, and 10 of them were included in the final analysis. The primary reasons for exclusion during full-text review included Outcome Relevance, Wrong comparison, and Study Design Incompatibility. The complete study selection process is illustrated in the PRISMA flow diagram (Figure 1).

### 3.2. Characteristics of Included Articles

A total of twelve studies (Table 1), comparing total hip replacement (THR) and hemiarthroplasty for displaced femoral neck fractures were included, spanning publications from 2006 to 2024. examined elderly patients with age ranges from over 50 years to over 80 years. Sample sizes varied widely, ranging from small cohorts of 20 patients to large databases including over 160,000 patients. Overall, the findings of most articles showed no major difference in venous thromboembolic risk between the two procedures, despite minor variations reported in a few studies.

### 3.3. Risk of VTE Between THR and HA

**Figure 2:**
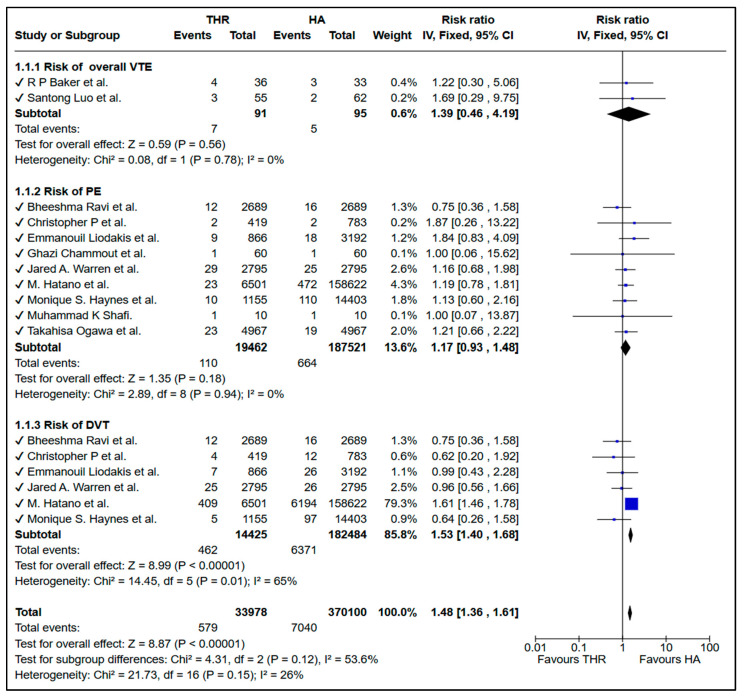
Forest plot comparing venous thromboembolic events between total hip replacement (THR) and hemiarthroplasty (HA) in patients with femoral neck fractures [3,9,10,11,12,13,14,15,16,18,19]. The blue squares represent the risk ratio (effect estimate) for each individual study, and the black diamonds represent the pooled (overall) risk ratio for each subgroup and for the total analysis.

A total of 33,978 patients undergoing total hip replacement (THR) and 370,100 undergoing hemiarthroplasty (HA) were included in the analysis. The pooled results demonstrated that THR was associated with a significantly higher overall risk of thromboembolic events compared to HA (RR = 1.48, 95% CI: 1.36–1.61, *p* < 0.00001; I^2^ = 26%) (Figure 2). Subgroup analyses showed no significant differences in the risk of overall venous thromboembolism (RR = 1.39, 95% CI: 0.46–4.19, *p* = 0.56) or pulmonary embolism (RR = 1.17, 95% CI: 0.93–1.48, *p* = 0.18), although the risk of deep vein thrombosis was significantly higher with THR (RR = 1.53, 95% CI: 1.40–1.68, *p* < 0.00001).

### 3.4. Risk of Cardiovascular Complications Between THR and HA

**Figure 3:**
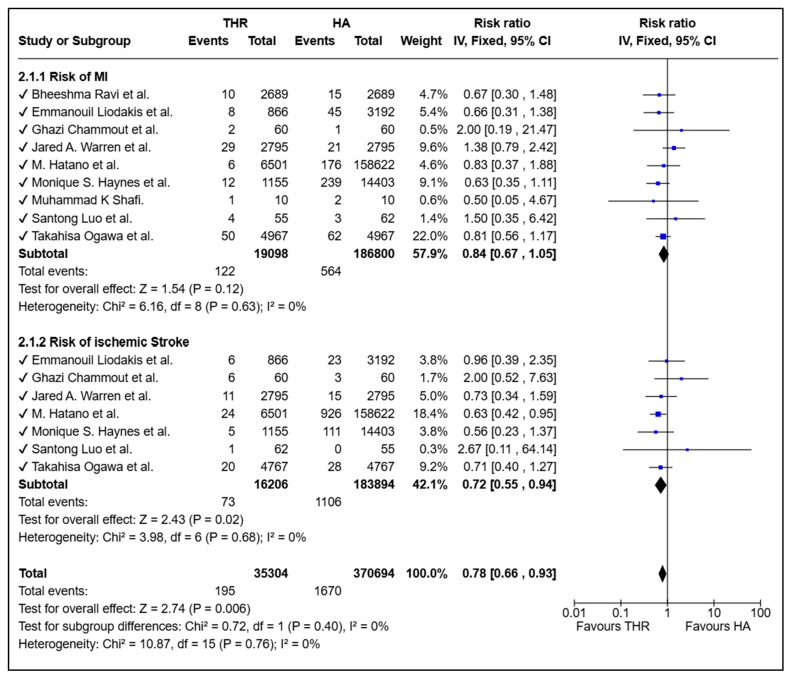
Forest plot comparing arterial thromboembolic events between total hip replacement (THR) and hemiarthroplasty (HA) in patients with femoral neck fractures [3,11,12,13,14,15,16,18]. The blue squares represent the risk ratio (effect estimate) for each individual study and the black diamonds represent the pooled (overall) risk ratio for each subgroup and for the total analysis.

In terms of cardiovascular outcomes, 35,304 THR patients and 370,694 HA patients were analyzed. The pooled risk of myocardial infarction did not differ significantly between groups (RR = 0.84, 95% CI: 0.67–1.05, *p* = 0.12; I^2^ = 0%). However, ischemic stroke risk was significantly lower among THR patients compared with HA (RR = 0.72, 95% CI: 0.55–0.94, *p* = 0.02), leading to an overall 22% reduction in combined cardiovascular risk with THR (RR = 0.78, 95% CI: 0.66–0.93, *p* = 0.006; I^2^ = 0%) (Figure 3).

### 3.5. Sensitivity Analysis

The DVT subgroup analysis demonstrated high heterogeneity (I^2^ = 65%). Given this substantial heterogeneity, we conducted a sensitivity analysis by removing the study by Hatano et al., which contributed the largest weight to the analysis (79.3%) and appeared to be a potential source of heterogeneity. After excluding this study, the association between THR and increased DVT risk remained statistically significant but was substantially attenuated (RR = 1.28, 95% CI: 1.12–1.47, *p* = 0.001), and heterogeneity was eliminated (I^2^ = 0%). This suggests that while the Hatano study contributed to both the magnitude of the effect and the observed heterogeneity, a modest increased DVT risk with THR persists even after excluding this large study.

### 3.6. Risk of Bias Assessment

Using the Newcastle–Ottawa Scale, most included studies demonstrated a low risk of bias, with scores ranging between 7 and 9 out of 9. Nearly all studies achieved full marks in the selection and outcome domains, indicating appropriate cohort selection, reliable exposure assessment, and clear outcome evaluation (Table 2). All included studies were assessed as having low or moderate risk of bias. Therefore, a sensitivity analysis based on exclusion of high-risk studies was not performed, as it would not alter the composition of the data set. For the randomized controlled trials assessed with the RoB 2 tool, Chammout et al. [12] showed a low risk of bias, whereas Baker et al. [9] had some concerns related to allocation concealment and outcome reporting. Overall, consistently high scores support a generally low risk of bias across studies.

### 3.7. Publication Bias

**Figure 4:**
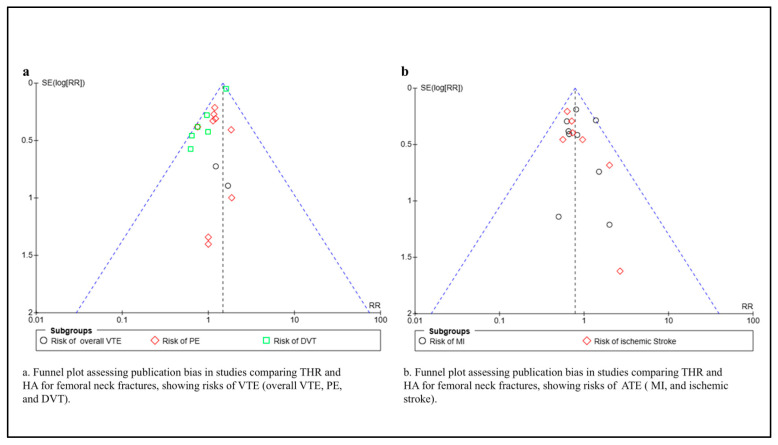
Funnel plots for assessment of publication bias: (**a**) venous thromboembolic outcomes; (**b**) arterial thromboembolic outcomes.

Funnel plot analysis (Figure 4) assessed potential publication bias in the included studies. For VTE outcomes (plot a), the distribution appears reasonably symmetric for DVT studies, though some asymmetry is evident for PE studies, with smaller studies clustering toward favoring one intervention. For arterial thromboembolism outcomes (plot b), while MI studies show relatively symmetric distribution, ischemic stroke studies demonstrate notable asymmetry, with smaller studies potentially favoring THR. This pattern suggests possible small-study effects or publication bias, particularly for stroke outcomes, which may limit the reliability of the observed protective effect of THR against ischemic stroke. However, the limited number of studies per outcome precluded formal statistical testing for publication bias.

## 4. Discussion

In this systematic review and meta-analysis, the results indicate that THR is linked to a significantly higher risk of deep vein thrombosis (DVT) and combined Venous thromboembolism (VTE) compared to hemiarthroplasty. In our meta-analysis, the pooled results initially demonstrated a statistically significant association, especially for the DVT subgroup. However, upon conducting a sensitivity analysis and removing the large, high-quality study by Hatano et al. [19], the pooled results remained statistically significant but with an attenuated effect size. This finding highlights the substantial influence this individual study exerts due to its large sample size and robust statistical approach, including the use of instrumental variable analysis. Importantly, this does not undermine the quality or validity of the study; rather, it reflects its disproportionate weighting within the meta-analysis and the sensitivity of the pooled estimate to its inclusion. This study was conducted with a robust design, a nationally representative patient sample, and sophisticated statistical adjustment for both measured and unmeasured confounders, making it a pivotal contribution to the evidence base. Considering these factors, we elected to retain it within our analysis. We clearly acknowledge its influence in our results, and interpret our findings with caution, highlighting the need for further studies with comparable methodological rigor and scale to confirm the observed effects. There was no significant difference in the rate of pulmonary embolism (PE) between the two procedures. Interestingly, THR was associated with a reduced risk of ischemic stroke, suggesting a possible lower cardiovascular risk.

Our findings provide a nuanced perspective on the thromboembolic and cardiovascular outcomes associated with THR and hemiarthroplasty in patients with femoral neck fractures. In our pooled analysis of over 160,000 hip procedures, for instance, Peng et al. [20], conducted a meta-analysis specifically focused on thromboembolic complications following THR and hemiarthroplasty. Their pooled analysis, showing no significant difference in overall thromboembolic events between the two procedures, aligns partially with our observation that aggregate VTE risk does not differ significantly with a pooled risk ratio of 1.27 (95% CI: 0.54–3.01; *p* = 0.44). However, by dissecting individual event types, our study reveals that THR drives up DVT/VTE incidences, an insight that Peng et al. [20] could not address due to the narrow scope of their analysis. Similarly, Li and Luo [21] presented a broader evaluation of perioperative outcomes following THR and hemiarthroplasty, analyzing data from 19 studies encompassing over 458,000 patients. Although their analysis did not primarily target thromboembolic or cardiovascular outcomes, it included key vascular complications, such as MI risk ratio 1.45 (95% CI: 0.98–2.15; *p* = 0.74). and PE risk ratio 1.16 (95% CI: 0.80–1.67; *p* = 0.39). This reveals no significant difference in the incidence of these events, aligning with our findings. However, our study further differentiates the vascular profile of each procedure, showing that while THR was linked to a higher incidence of VTE, it was also associated with a reduced risk of ischemic stroke and other major cardiovascular events. These findings build upon and refine the work of Li and Luo, highlighting the need for a comprehensive evaluation of vascular outcomes when selecting the most appropriate surgical option for femoral neck fractures. Another meta-analysis comparing the two procedures for displaced femoral neck fractures found no significant difference in thromboembolic risk, which was assessed as part of general perioperative complications (including thromboembolism, pneumonia, urinary tract infections, and cardiovascular events). General complications occurred in (21.7%) of THR patients and (19.4%) of hemiarthroplasty patients, with a pooled risk ratio of 1.15 (95% CI: 0.91–1.45; *p* = 0.25), indicating no statistically significant difference between the two procedures as mentioned by Yu L et al. [22] Building on insight from an RCT conducted by Blomfeldt et al. [23], out of 120 hip procedures, only one patient (1.7%) in the hemiarthroplasty cohort developed DVT, and none in the THR group, with no PE events reported over four months. By contrast, our large-scale meta-analysis reveals a substantially higher DVT risk following THR, while PE rates remain statistically indistinguishable. While overall thromboembolic risk may appear similar between THR and Hemiarthroplasty, the distribution of event types varies meaningfully.

### Strengths and Limitations

This article has several strengths that enhance the reliability of the findings. First, the study adhered to PRISMA guidelines and was prospectively registered in PROSPERO, ensuring a transparent and methodologically sound approach. A comprehensive literature search was conducted across multiple databases, including both randomized controlled trials and large-scale cohort studies, which contributed to the breadth and applicability of the results. In addition, the methodological quality of the included studies was assessed using standardized tools such as the Newcastle–Ottawa Scale (NOS) and RoB 2, which confirmed that the overall risk of bias was low.

However, some limitations should be acknowledged. There was notable variation among the included studies in terms of design, sample size, and follow-up duration, which may limit the generalizability of the findings. A substantial heterogeneity was observed across the included studies, primarily driven by the study conducted by Hatano et al., which had the largest sample size. The pooled effect within the DVT subgroup was predominantly influenced by the study of Hatano et al., which contributed a disproportionate statistical weight. Sensitivity analysis confirmed that excluding this study reduced heterogeneity (I^2^ from 65% to 0%) without altering the direction of association. This indicates that while the overall trend is consistent, the magnitude of the pooled estimate largely reflects the influence of this single, high-weight study. Accordingly, the findings should be interpreted with caution, as the overall effect size and heterogeneity were largely driven by Hatano et al. The disproportionate contribution of this study inflated the magnitude of observed effect and study heterogeneity, which may limit the generalizability of the summary risk ratio.

Despite applying strict inclusion criteria, the dominance of retrospective cohort studies introduced inherent weaknesses, such as potential selection bias and inconsistencies in reporting critical clinical details, including thromboprophylaxis strategies and patient mobility. Furthermore, a significant selection bias existed within the THR group. Patients undergoing THR are typically healthier and have fewer comorbidities than non-surgical patients. Consequently, the lower observed risk of ATE or ischemic stroke may reflect patient selection rather than a true causal effect.

An additional limitation of this study is the lack of data on time-to-surgery, which is a key confounder in femoral-neck fracture cohorts since prolonged waiting periods are associated with increased DVT risk. The inability to account for this variable may have influenced the observed association between THA and VTE. Therefore, this relationship should be interpreted with caution, as causal inferences cannot be firmly established. Surgeons should carefully evaluate patients’ risk factors before selecting the type of surgery, as THR is associated with a higher risk of DVT. Given the increased risk of THR, aggressive thromboembolism prophylaxis, including anticoagulant therapy and mechanical compression, should be implemented for patients undergoing this procedure. Additionally, specific guidelines should be developed to outline strategies for reducing thrombotic events in patients undergoing THR and for monitoring these patients postoperatively to prevent further complications.

Regarding research implications, there is a need for high-quality randomized controlled trials to further validate the association between THR and thrombotic events. Additional studies with similar methodological rigor and larger sample sizes are required to validate these findings, and studies should also investigate how patient-specific factors such as age, gender, obesity, and existing comorbidities influence the occurrence of thrombotic events after THR.

## 5. Conclusions

This systematic review and meta-analysis evaluated thromboembolic and cardiovascular complications associated with THR versus hemiarthroplasty in patients with femoral neck fractures. THR was associated with a 53% increased risk of deep vein thrombosis (RR = 1.53) and a 48% increased risk of combined venous thromboembolism (RR = 1.48) compared to hemiarthroplasty. In contrast, hemiarthroplasty was linked to a higher risk of ischemic stroke and combined arterial thromboembolic events. These findings suggest that while THR may carry a greater venous thrombotic burden, hemiarthroplasty may pose a higher arterial risk. Surgical decision-making should therefore be individualized, considering patient comorbidities, functional expectations, and the distinct thrombotic profiles of each procedure. Future prospective randomized controlled trials are needed to control thromboprophylaxis regimens, assess long-term cardiovascular outcomes, and better delineate patient subgroups that may benefit most from either intervention.

## Figures and Tables

**Table 1:** Characteristics included articles (year of publication, study design, sample size, age range, main outcomes, risk of VTE.

Study (Author, Year)	Study Design	Sample Size (THR/HA)	Age Range	Main Outcomes	VTE Risk
Baker et al. (2006) [9]	Randomized Controlled Trial (RCT)	(40/41)	>60 years	Better hip scores and walking in THR; more acetabular erosion in HA	PE and DVT RISK is higher in HA group
Miller et al. [10] (2014)	Retrospective cohort	(419/783)	>50 years	Shorter hospital stays; minor complications lower in THR	No major difference
Liodakis et al. [11] (2016)	Retrospective cohort	(866/3192)	≥70 years	No major complications difference; higher transfusion in THR	No major difference
Chammout et al. [12] (2019)	Prospective Randomized Controlled Trial (RCT)	(60/60)	≥80 years	No difference in hip function, QoL, or complications	No major difference
Warren et al. [13] (2019)	Retrospective matched cohort	(2795/2795)	≥65 years	Lower mortality, more home discharges in THR	No major difference
Ravi et al. [14] (2019)	Retrospective matched cohort	(2689/2689)	≥60 years	Lower revision rate, higher dislocation in THR	No major difference
Haynes et al. [15] (2020)	Retrospective cohort	(1155 /14,403)	≥70 years	30-day outcomes (mortality, transfusion)	No major difference
Ogawa et al. [16] (2020)	Retrospective matched cohort	(4967/4967)	≥60 years	Higher dislocation and revision in THR	No major difference
Suarez et al. [17] (2020)	Retrospective cohort	(4124/12089)	≥50 years	THR has had fewer major complications recently	No major difference
Shafi et al. [18] (2022)	Retrospective cohort	(10/10)	>60 years	Fewer complications in THR	No major difference
Luo et al. [3] (2023)	Retrospective cohort	(55/62)	>75 years	Better 5-year function and QoL in THR	No major difference
Hatano et al. [19] (2024)	Retrospective cohort	(6501/158,622)	>60 years	Lower mortality in THR	Higher DVT in THR

DVT: Deep Venous Thrombosis, PE: Pulmonary Embolism, THR: Total Hip Replacement, HA: Hemiarthroplasty, Qol: Quality of Life.

**Table 2:** Risk of bias assessment using the Newcastle-–Ottawa scale.

Study	Selection				Comparability		Outcome			Total (9/9)
	Representative of the Exposed Cohort	Selection of the External Control	Ascertainment of Exposure	Outcome of Interest Does Not Present at the Start of the Study	Comparability of Cohorts		Assessment of Outcomes	Sufficient Follow-Up Time	Adequacy of Follow-Up	
					Main Factor	Additional Factor				
Miller et al. 2015 [10]	*	*	*	*	*	*	*	*	0	(8/9)
Liodakis et al. 2016 [11]	*	*	*	*	*	*	*	0	*	(8/9)
Ravi et al. 2019 [14]	*	*	*	*	*	*	*	*	*	(9/9)
Warren et al. 2019 [13]	*	*	*	*	*	*	*	*	*	(9/9)
Ogawa et al. 2020 [16]	*	*	*	*	*	*	*	*	*	(9/9)
Suarez et al. 2020 [17]	*	*	*	*	*	*	*	0	*	(8/9)
Haynes et al. 2020 [15]	*	*	*	*	*	*	*	*	*	(9/9)
Shafi et al. 2022 [18]	0	*	*	*	*	0	*	*	*	(7/9)
Luo et al. 2023 [3]	*	*	*	*	*	0	*	*	0	(7/9)
Hatano et al. 2024 [19]	*	*	*	*	*	*	*	*	*	(9/9)

Yes = *, No = 0, Total score.

## Data Availability

The data supporting the findings of this study are available from the corresponding author upon reasonable request.

## Abbreviations

The following abbreviations are used in this manuscript:

THR	Total Hip Replacement
HA	Hemiarthroplasty
VTE	Venous Thromboembolism
DVT	Deep Vein Thrombosis
PE	Pulmonary Embolism
RoB 2	Risk of Bias 2 (Tool)
